# Malposition of subclavian vein catheter inserted through indirect technique in a pediatric liver transplantation: a case report

**DOI:** 10.1186/1757-1626-2-7998

**Published:** 2009-06-03

**Authors:** Demet Coskun, Ahmet Mahli, Sema Oncul, Gizem Ilvan, Aydin Dalgic

**Affiliations:** 1 Department of Anesthesia and Reanimation, Gazi University Medical FacultyAnkaraTurkey; 2 Transplantation Unit, Gazi University Medical FacultyAnkaraTurkey

## Abstract

**Introduction:**

Clinicians use either direct or indirect (Seldinger) techniques for internal juguler or subclavian vein catheterization. This report aims to point out that the success rate of the direct technique where the catheter is inserted directly through the cannula may be higher particularly in catheterization of pediatric cases.

**Case presentation:**

A 7.5-month-old female infant weighing 7200 gm was operated on for liver transplantation. The patient suffered jaundice at one month of age and was diagnosed with neonatal colestatic hepatitis. After routine monitoring, via indirect technique, central catheterization was attempted through internal jugular vein. However, the attempt failed. Therefore, again via indirect technique, catheterization was achieved through the right subclavian vein and fixed at 8 cm. After the operation started, fluid replacement and central venous pressure monitoring were performed with this catheter. Immediately after the operation, a control postero-anterior chest radiograph of the patient was obtained. This graph revealed that the tip of the catheter was fixed in the right internal jugular vein. Since the vital symptoms of the patient were not stable, the catheter was not removed and fluid replacement was performed via this technique. The catheter was removed on the postoperative 2^nd^ day.

**Conclusion:**

The J wire advanced via the indirect technique advances anatomically following the upper wall of subclavian vein. Because of the smaller vessel dimensions and sharper, more angulated routes the subclavian and internal jugular veins make in infants, the rigid J wire may advance in the cephalic direction. However, in the technique where the catheter (Cavafix ^®^ catheter) is inserted directly through the cannula, this probability is less since J wire is not used and the catheter employed is flexible. We concluded that especially in pediatric cases, employment of the technique where the catheter is inserted directly through the cannula would be more convenient in order to decrease the catheter malpositioning probability.

## Introduction

The most common complication of central venous catheter insertion is malpositioning, accounting for 14-81% of all associated complications [1,2]. Malpositioning creates a potential for mistakes in diagnosis and follow-up of patients as well as for severe complications [3].

In this report, the case of a 7.5-month-old infant with a malpositioned central venous catheter (CVC) is presented. Insertion of a CVC into the right subclavian vein was attempted via indirect (Seldinger) technique, but it was inserted into the right internal jugular vein by mistake. So, this report aims to point out that the success rate of the technique where the catheter is placed directly through the cannula may be higher particularly in catheterization of pediatric cases.

## Case presentation

**Figure 1.**
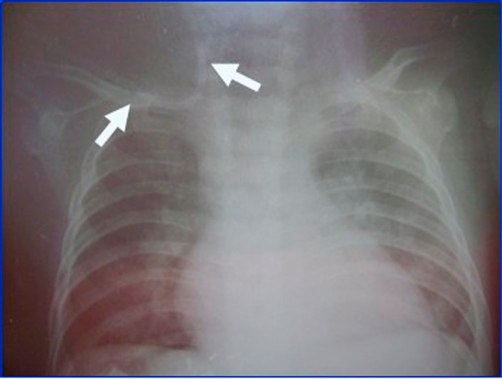
Malposition of the subclavian catheter.

A 7.5-month-old Turkish female infant weighing 7200 gm was operated on for liver transplantation. The patient suffered jaundice at one month of age and was diagnosed with neonatal colestatic hepatitis. After routine monitoring, the neck and infraclavicular region were cleaned with antiseptic solution, and via indirect (Seldinger) technique, central catheterization was attempted through internal jugular vein. However, the attempt failed. Therefore, again via indirect technique, catheterization was achieved through the right subclavian vein (Arrow Central Venous Catheter, 16 G, 20 cm, 0.32 inch). No arrhythmia was observed on the monitor while J wire was advanced. After blood aspiration, the catheter was fixed at 8 cm. After the operation started, fluid replacement and central venous pressure (CVP) monitoring were performed with this catheter. The CVP values ranged between 7-13 mmHg throughout the operation. The operation lasted for 12 hours, and immediately after the operation, a control postero-anterior chest radiograph of the patient was obtained. This graph revealed that the tip of the catheter was fixed in the right internal jugular vein (Figure 1). Since the vital symptoms of the patient were not stable, the catheter was not removed and fluid replacement was performed via this technique. The catheter was removed on the postoperative 2^nd^ day.

## Discussion

Central venous catheter insertion is a common procedure used in monitoring CVP, administration of some drugs, blood and blood products, antineoplastic treatment, parenteral nutrition, and bone marrow transplantation. Central venous catheters can be centrally or peripherally inserted; however, the commonly preferred technique is the internal jugular or subclavian veins [4]. The incidence rate for catheter-borne or procedure related complications in these practices is 1-42%. These complications can be listed as arterial puncture, pneumothorax, chylothorax, vein and nerve damage, infection, thrombosis, malposition, folding of the catheter, hemothorax, cardiac tamponade, air embolism, arrhythmia, and death [1,3,5].

**Figure 2.**
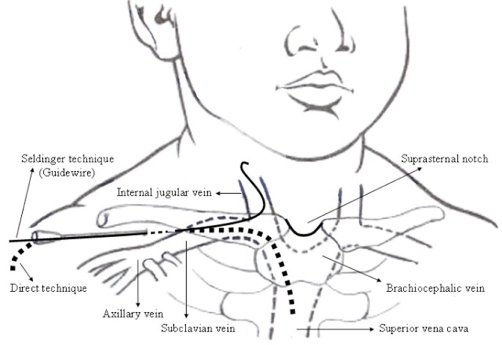
Anatomy of the subclavian and jugular veins in pediatric age group.

In pediatric patients, CVCs are necessary for surgical or medical reasons. These catheters are used in monitoring CVP as well as for short and long-term feeding. Although uses of CVCs in the children are similar to the adult patient, attaining central venous access in the young child is more difficult because of the smaller vessel dimensions and sharper, more angulated routes the subclavian and internal jugular veins make in infants [6] (Figure 2).

In central venous catheterization procedures, the tip of the catheter should be located right above the region where the superior vena cava and the right atrium merge. On a control chest radiograph, the most proper location for the catheter tip is considered to be 2 cm proximal to the pericardial line [7]. As the tip can not directly be seen during the procedure, malposition of the CVC is not a rare complication [1,8]. Janik et al reported a rate of 7.3% for this complication in children under the age of five [3]. The most common reason for early dysfunction of the catheter is the improper location of the catheter tip, which can be determined through radiography [9].

Malpositioning of CVC is more common in subclavian approach. Some studies have reported a slightly higher risk of malposition in the approach through the right subclavian vein compared to the left subclavian vein, although others found no such difference [3]. More commonly, malpositioning is towards the internal jugular vein of the same side. However, the catheter tip is rarely inserted into brachiocephalic, azigos, superior intercostal veins, or the internal and subclavian veins of the opposite side [9]. This may result in mismeasurement of CVP, cloth formation, catheter erosion, and increased risk for chemical and bacterial thrombophlebitis. Central venous catheter procedure may fail even in the most experienced hands. In addition, numerous attempts of the intervention increases the risk of complications [5]. Electrocardiography, ultrasonography, and internal jugular vein occlusion tests can be used to insert the catheter into the proper place [10,11]. Guth [2] asserts that in properly conducted catheterization procedures, no radiographic evaluations are needed unless a complication is suspected, while some authors advocate routine use of control chest radiograph because malpositioning and pneumothorax can be detected by radiography obtained immediately after the procedure [1,4]. In our patient, central venous catheterization was performed just before the operation and no complications were suspected; thus, no intraoperative radiographic evaluation was needed.

## Conclusion

The J wire advanced via the indirect technique advances anatomically following the upper wall of subclavian vein. Because of the smaller vessel dimensions and sharper, more angulated routes the subclavian and internal jugular veins make in infants, the rigid J wire may advance in the cephalic direction. However, in the technique where the catheter (Cavafix® catheter) is inserted directly through the cannula, this probability is less since J wire is not used and the catheter employed is flexible. We concluded that especially in pediatric cases, employment of the technique where the catheter is inserted directly through the cannula would be more convenient in order to decrease the catheter malpositioning probability.

## List of abbreviations

CVC: Central venous catheter
CVP: Central venous pressure
